# Protective reactive thymus hyperplasia in COVID-19 acute respiratory distress syndrome

**DOI:** 10.1186/s13054-020-03440-1

**Published:** 2021-01-04

**Authors:** Pelagia Cuvelier, Hélène Roux, Anne Couëdel-Courteille, Jacques Dutrieux, Cécile Naudin, Bénédicte Charmeteau de Muylder, Rémi Cheynier, Pierre Squara, Stefano Marullo

**Affiliations:** 1 Clinique Ambroise Paré, 27 bd Victor Hugo, 92200 Neuilly-sur-Seine, France; 2 Université de Paris, CNRS, INSERM, Institut Cochin, 75014 Paris, France

**Keywords:** COVID-19, Thymus reactivation, TREC

## Abstract

**Background:**

Patients with COVID-19 (COVID) may develop acute respiratory distress syndrome with or without sepsis, coagulopathy and visceral damage. While chest CT scans are routinely performed in the initial assessment of patients with severe pulmonary forms, thymus involvement and reactivation have not been investigated so far.

**Methods:**

In this observational study, we systematically scored the enlargement of the thymus and the lung involvement, using CT scans, in all adult patients admitted to the ICU for COVID or any other cause (control group) at one centre between March and April 2020. Initial biological investigations included nasal detection of SARS-CoV-2 ribonucleic acid by polymerase chain reaction (PCR). In a subgroup of 24 patients with different degrees of pulmonary involvement and thymus hypertrophy, plasma cytokine concentrations were measured and the export of mature T cells from the thymus was estimated simultaneously by PCR quantification of T cell receptor excision circles (TRECs).

**Results:**

Eighty-seven patients were studied: 50 COVID patients and 37 controls. Non-atrophic or enlarged thymus was more commonly observed in COVID patients than in controls (66% vs. 24%, *p* < 0.0001). Thymus enlargement in COVID patients was associated with more extensive lung injury score on CT scans (4 [3–5] vs. 2 [1.5–4], *p* = 0.01), but a lower mortality rate (8.6% vs. 41.2%, *p* < 0.001). Other factors associated with mortality were age, lymphopaenia, high CRP and co-morbidities. COVID patients had higher concentrations of IL-7 (6.00 [3.72–9.25] vs. 2.17 [1.76–4.4] pg/mL; *p* = 0.04) and higher thymic production of new lymphocytes (sj/βTREC ratio = 2.88 [1.98–4.51] vs. 0.23 [0.15–0.60]; *p* = 0.004). Thymic production was also correlated with the CT scan thymic score (*r* = 0.38, *p* = 0.03) and inversely correlated with the number of lymphocytes (*r* = 0.56, *p* = 0.007).

**Conclusion:**

In COVID patients, thymus enlargement was frequent and associated with increased T lymphocyte production, which appears to be a beneficial adaptation to virus-induced lymphopaenia. The lack of thymic activity/reactivation in older SARS-CoV-2 infected patients could contribute to a worse prognosis.
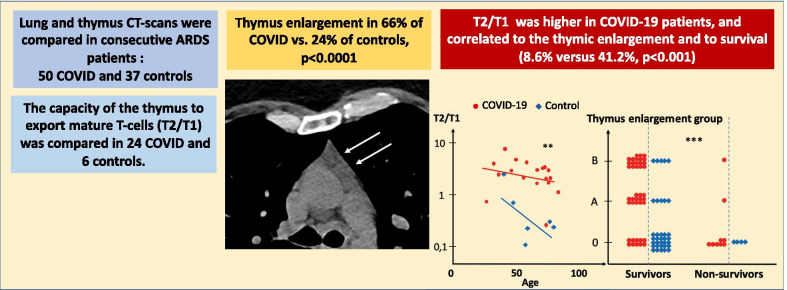

## Introduction

Less than one year after the beginning of the coronavirus 2019 pandemic (COVID) due to the virus identified as SARS-CoV-2, an increasing number of studies has markedly improved our knowledge of its epidemiology and illustrated the large spectrum of clinical consequences of the infection. Although a large proportion of infected people remains asymptomatic or develops a more or less severe flu-like syndrome, some patients can develop severe forms and require hospitalisation [1, 2]. In France, the infection fatality rate ranges from 0.001% in individuals under 20 years of age to 8.3% in patients over 80 years old [3].

Several studies have identified clinical and biological risk factors for death, which include age, gender, co-morbidities (such as obesity, diabetes, hypertension), D-dimer concentration, low lymphocyte counts and high C-reactive protein [2, 4]. Recent investigations have highlighted the role of increased pro-inflammatory cytokines (cytokine storm) [5, 6], impaired type I interferon responses [7], profoundly altered T cell phenotypes [8] and functional exhaustion of antiviral lymphocytes [9, 10] in the severity of COVID. These findings, similar to those reported in previous pathogenic human coronavirus epidemics due to SARS-CoV-1 and Middle East respiratory syndrome coronavirus (MERS-CoV), reflect the shift from a protective regulated inflammatory response against the virus to pathogenic dysregulated inflammation [11].

In this context, during the initial chest CT scan assessment of adult patients admitted to our intensive care unit (ICU) for COVID, we noticed a previously unreported marked thymus enlargement in many individuals. Although the thymus continues to generate new T lymphocytes in adults, it undergoes progressive physiological involution with age [12, 13]. Enlargement of the thymus region, especially in the elderly, is mostly observed in autoimmune conditions and tumours, or in response to profound lymphopaenia [14], whether or not associated with sepsis. Such lymphopaenia is frequently observed in patients with severe COVID [15]. However, thymic volume is not directly related to the extent of thymic function, which consists of producing and exporting mature T cells into the blood. Accordingly, the most accurate measurement of thymic activity in humans relies on the quantification of T cell receptor excision circles (TRECs), which are by-products of chromosomal rearrangements that occur at T cell receptor loci (TCRA and TCRB, leading to sjTREC and βTRECs, respectively) during T cell development in the thymus. The ratio of both types of TRECs (sj/βTREC), quantified in circulating lymphocytes, is considered to be a surrogate marker of the extent of thymic production of new T cells [16, 17].

The goal of this study was to quantify the enlargement of the thymus in all chest CT scans performed in patients admitted to our intensive care unit in March and April 2020, and to compare the CT scan scores of COVID patients with those of patients hospitalised for other reasons. The clinical condition of the patients, their thymic function and outcomes were then examined according to these scores.

## Methods

### Patients

This observational study was conducted in all 87 adult patients (50 COVID and 37 controls) hospitalised in the intensive care unit (ICU) of the Clinique Ambroise Paré (Neuilly, France) from the beginning of March to the end of April 2020. During this time period, this ICU, which under normal circumstances principally treats patients admitted for cardiovascular diseases, contributed to the national French program aimed at enhancing the hospitalisation capacity in the ICU for severely affected SARS-CoV-2-infected patients.

According to French national regulations, systematic written informed consent was requested from all patients upon admission, for the potential anonymous use in clinical research studies of clinical and paraclinical data obtained during hospitalisation.

### Thoracic CT scans

**Fig. 1:**
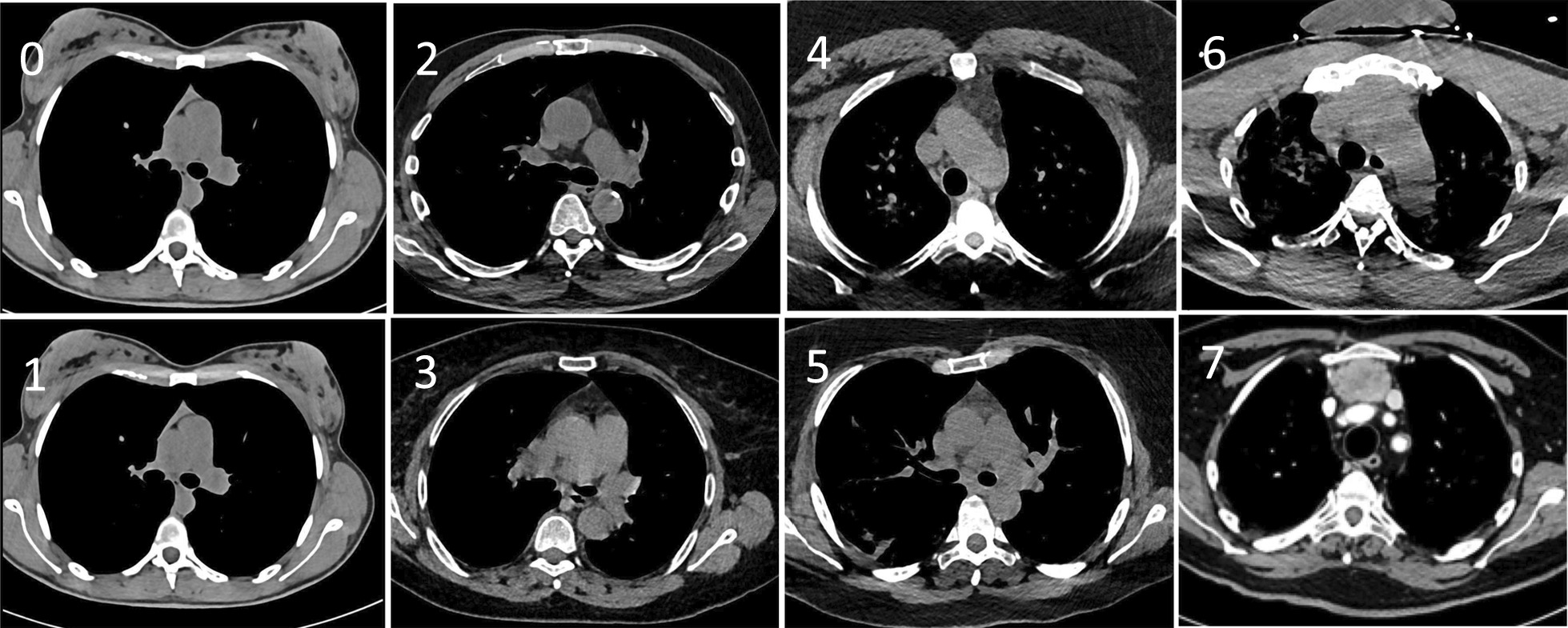
Chest CT scan images. Representative chest CT scan images of the thymic area of patients hospitalised for COVID, illustrating the classification of thymus aspects depicted in Table 2. Arrows indicate the different types of thymus hyperplasia

All patients had CT scans at admission. Thoracic CT scans were performed for the initial evaluation of pulmonary conditions upon admission. Specific settings were used to examine the thymus area. All images were independently reviewed and classified by two radiologists. In case of disagreement, they reviewed the scans together and were asked to give a final common classification. The classification of pulmonary parenchymal images and of thymus aspects is shown in Tables 1 and 2 as well as in Fig. 1.

**Table 1:** CT scan classification of COVID-associated pneumopathy

0	Absent or minor pulmonary parenchymal changes
1	Limited ground-glass opacities
2	Bilateral ground-glass opacities < 50% of pulmonary parenchyma
3	Idem 2, with superimposed inter/intra lobular septal thickening, i.e. ‘crazy paving’
4	Bilateral ground-glass opacities > 50% of pulmonary parenchyma
5	Idem 4, with superimposed ‘crazy paving’
6	Idem 5, with pulmonary fibrosis

**Table 2:** CT scan classification of thymus aspects

0	0	Fatty thymus atrophy (the most common in middle aged adults)
A	1	Homogeneous non-fatty thymus (common in young adults)
	2	Fat in the thymus area associated with micronodules
	3	Moderate infiltration of the thymus area
B	4	Hyperplasia, marked infiltration, micronodules
	4	Hyperplasia with well-defined contours
	6	Nodular hyperplasia without a tumour mass
	7	Pseudo-tumoral mass with well-defined contours

### Laboratory procedures

Initial clinical laboratory investigations included a complete blood count, serum biochemical tests including liver and kidney function, creatine kinase, lactate dehydrogenase, electrolytes, fibrinogen, C reactive protein (CRP) and a coagulation profile. SARS-CoV-2 infection was confirmed by nasal viral RNA detection using reverse transcriptase polymerase chain reaction (RT-PCR) following the procedure recommended by the manufacturer and/or clinical history associated with the notion of plausible contamination by a proven infected contact.

Plasma concentrations of 23 cytokines (IL-1β, IL-4, IL-6, IL-7, IL-8, IL-10, IL-12p70, IL-15, IL-17A, IL-17E, IL-17F, IL-21, IL-22, IL-23, IL-33, TSLP, GM-CSF, TNFα, MDC, MIP-1α, MIP-1β, MIP-3β and SDF-1) were measured on the same day in 24 hospitalised patients, including 18 COVID patients and 6 control individuals with different degrees of pulmonary involvement and thymus hypertrophy, using a multiplex ELISA (U-plex technique; Meso Scale Diagnostics, Rockville, Maryland) according to the manufacturer’s instructions.

The capacity of the thymus to export mature T cells into the bloodstream was estimated in the same patients through PCR quantification of two types of T cell receptor excision circles (sjTRECs and βTRECs) in peripheral blood mononuclear cells and by the calculation of the sj/βTREC ratio, a good estimate of the thymic output ratio, as described previously [16–18].

### Statistical analysis

Descriptive statistics were used to present baseline characteristics for the total population and for the various groups. The normality of continuous data was assessed using the Shapiro–Wilk test. Continuous variables are presented as mean ± SD or median [25–75% interquartile range], according to their distribution, and categorical variables are presented as the number of patients in each category and the corresponding percentages. Missing values concerned four patients for leukocyte counts, two patients for haemoglobin levels, one patient for CRP, and 34 patients for D-dimer and fibrinogen. They were not replaced and were considered as empty cells in all calculations. Quantitative variables were compared using Student’s *t* test or the Mann–Whitney *U* test according to the normality of the distribution. Categorical variables were compared using Pearson tests. Kruskal–Wallis tests were used to compare three groups. Bivariate (correlation coefficient) analyses were performed to determine the relationships between the variables. The Bonferroni correction was used for multiple comparison adjustments. JMP software was used for the statistical analysis, and a value of *p* < 0.05 was considered to be statistically significant.

## Results

Among the 87 patients enrolled in the study, 37 were considered as non-infected by SARS-CoV-2 (control group). Fifty patients had confirmed COVID (COVID group). The control group (non-COVID) was composed of patients admitted during the same period of time for other pathologies and who had a CT scan: medical cardiology (16), cardiac surgery (11), urology (5) and other (5). They had no clinical or radiological signs of COVID, and their PCR test was negative. A descriptive analysis of the two groups at the time of the first CT scan is summarised in Table 3.

**Table 3:** Descriptive analysis of the investigated patients

Thymus enlargement	Control (*n* = 37)				COVID (*n* = 50)			
	All (*n* = 37)	0 (*n* = 28)	A (*n* = 4)	B (*n* = 5)	All (*n* = 50)	0 (*n* = 17)	A (*n* = 14)	B (*n* = 19)
Demographics								
Sex ratio (M)	27 (73)	21 (75)	3 (75)	3 (60)	37 (74)	11 (65)	12 (86)	14 (74)
Age (years)	66.8 ± 14.4	64.3 ± 13.9	79.5 ± 13.1	71.2 ± 13.8	63.2 ± 16.5	75.4 ± 12.6	59.3 ± 12.7	55.3 ± 16.2***
BMI (kg/m^2^)	25.3 ± 4.1	25.7 [22.5–27.5]	27.1 [23.5–30.4]	23.3 [20.7–32.5]	28.8 ± 6.4	26.8 [25.0–30.3]	26.7 [24.2–29.8]	29.3 [26.0–35.7]
Medical history								
Hypertension	16 (43)	13 (46)	1 (25)	2 (40)	26 (52)	13 (76)	5 (36)	8 (42)*
Diabetes	3(8))	3 (11)	–	1 (20)	11 (22)	3 (18)	3 (21)	5 (26)
COPB	6 (14)	4 (14)	1 (25)	1 (20)	7 (14)	5 (29)	1 (7)	1 (5)
Renal failure	3 (11)	3 (11)	1 (25)	–	11 (22)	9 (53)	1 (7)	1 (5)**
Cancer	6 (16)	6 (21)	1 (25)	3 (60)	5 (10)	2 (12)	1 (7)	2 (11)
Smoking status								
Never	28 (76)	21 (75)	3 (75)	4 (80)	40 (80)	14 (82)	11 (79)	15 (79)
Former	3 (8)	3 (11)	–	–	9 (18)	3 (18)	3 (21)	3 (16)
Current	6 (16)	4 (14)	1 (25)	1 (20)	1 (2)	–	–	1 (5)
Biological data at admission								
Haemoglobin (g/L)	11.8 ± 2.6	12.0 ± 2.8	12.0 ± 1.9	10.2 ± 1.6	11.8 ± 2.0	11.1 ± 2.1	13.1 ± 1.7	11.5 ± 1.7
Leukocytes (× 10^9^/L	9.4 ± 3.6	8.7 [7.1–10.0]	11.0 [9.3–13.4]	8.1 [7.4–12.7]	7.5 ± 4.0	5.6 [3.9–8.9]	6.9 [5.4–8.1]	8.5 [5.1–10.1]
Lymphocytes (× 10^9^/L)	1.6 ± 1.4	1.2 [0.8–1.7]	1.3 [0.9–2.4]	1.1 [0.4–5.2]	0.6 ± 1.0	0.6 [0.5–1.5]	0.8 [0.7–1.0]	1.0 [0.5–1.5]
Monocytes (× 10^9^/L)	0.8 ± 0.4	0.8 [0.5–1.0]	0.9 [0.7–1.2]	0.5 [0.3–0.9]	0.6 ± 0.3	0.5 [0.3–0.9]	0.5 [0.3–0.7]	0.7 [0.3–0.9]
Lymphocytes (%)	17.4 ± 11.8	14.3 [9.8–23.2]	10.3 [9.5–21.8]	16.7 [4.3–40.7]	15.0 ± 8.5	15.0 [7.1–24.9]	12.2 [10.1–18.0]	13.1 [7.2–19.6]
Monocytes (%)	8.6 ± 3.0	9.1 [7.2–10.8]	8.6 [6.0–10.4]	5.9 [2.5–11.3]	9.9 ± 9.0	10.0 [7.3–11.4]	8.2 [5.8–9.1]	7.6 [5.5–10.7]
Fibrinogen (g/L)	4.9 ± 2.1	5.1 [2.7–6.0]	6.2 [5.9–6.5]	4.6 [2.0–7.1]	6.5 ± 1.9	4.8 [4.3–10.0]	5.9 [5.8–7.0]	6.9 [5.8–7.3]
D-dimer (ng/mL)	2217 ± 2016	1527 [1213–4240]	816 [521–1111]	2705	2900 ± 6500	1092 [814–1813]	1066 [878–2250]	1256 [825–2040]
CRP (mg/L)	57 ± 88	21 [2–67]	55 [19–79]	44 [11–113]	131 ± 104	128 [62–218]	115 [64–180]	86 [33–220]
Clinical data								
SAPS score	25 [20–38]	22 [19–30]	37 [32–50]	41 [21–59]	29 [24–36]	30 [24–36]	33 [25–39]	27 [18–32]
Mechanical ventilation	16 (43)	12 (43)	–	4 (80)	20 (40)	6 (35)	5 (36)	9 (45)
Length of stay (days)	14 ± 12	14 ± 12	4 ± 3	16 ± 14	12 ± 12	12 ± 12	17 ± 13	14 ± 10
Mech. ventilation (days)	0.7 ± 2.2	0.7 ± 2.2	–	1.8 ± 2.4	11.1 ± 3.2	11.1 ± 3.2	15.4 ± 3.8	5.2 ± 2.8
Secondary pneumopathy	–	–	–	–	6 (12)	2 (12)	2 (14)	2 (11)
Deceased	3 (8)	3 (11)	–	–	9 (18)	7 (41)	1 (7)	1 (5)**

BMI, body mass index; COPD chronic obstructive pulmonary disease; CRP, C-reactive protein. Mechanical ventilation indicates ventilation with orotracheal intubation. Secondary pneumopathy was defined as impaired ventilation and purulent aspiration with positive culture of endotracheal aspirate ≥ 10^5^ CFU/ml

^*^, **, ***p < 0.05, 0.001, 0.0001, respectively. Due to Bonferroni’s correction, significance was reached when *p* < 0.003

### Thymus enlargement characterises COVID patients

**Fig. 2:**
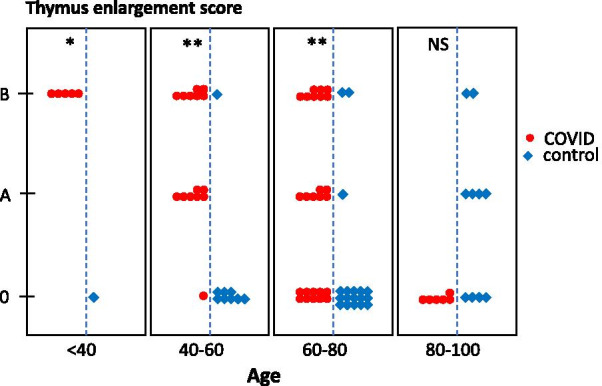
Thymic enlargement in COVID and control groups according to age categories. Thymic scores were determined through CT scan imaging in COVID (*n* = 50, red symbols) and COVID (*n* = 37, blue symbols) patients and displayed as a function of age categories. *p < 0.05; **p < 0.001, NS, not significant

The thymus aspects were classified into three groups (Table 2). Seventeen patients displayed fatty atrophy of the thymus (the most common in senior adults) and were classified as subgroup 0. Fourteen patients with a homogeneous non-fatty thymus, commonly observed in young adults, or a fatty thymus area associated with micronodules, were classified as subgroup A. Nineteen patients showing various levels of thymus hyperplasia or a pseudo-tumoral thymus represented subgroup B. Thymus persistence (subgroup A) or enlargement (subgroup B) was more frequent in the COVID group than in the control group (66% vs. 24%, *p* < 0.0001), except in patients over 80 years of age (Fig. 2, *p* < 0.05).

**Fig. 3:**
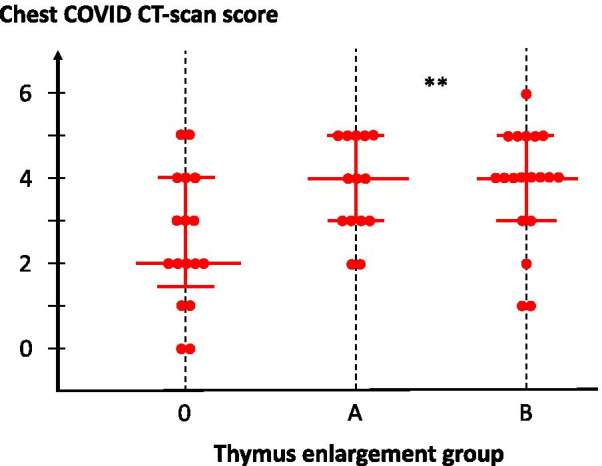
Lung damage in COVID patients, according to thymus enlargement group. Lung CT scan score is presented for each patient, according to individual thymus CT scan. Each dot represents an individual patient. Median values with the 25–75% interquartile ranges are shown; **p = 0.01 between groups

**Fig. 4:**
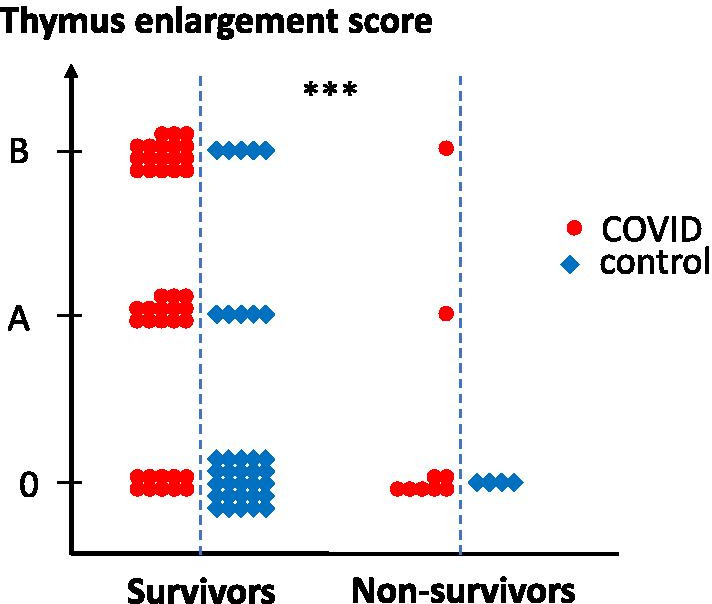
Survival as a function of thymic CT scan score. Thymus CT scan score is presented for each patient in survivor and non-survivor groups. Statistical differences between groups are shown on top; ***p < 0.0001

COVID patients with a persistent or enlarged thymus were younger than patients with normal thymic fatty atrophy (75.4 ± 12.6 years in subgroup 0 as compared to 59.3 ± 12.7 years and 55.3 ± 16.2 years in subgroups A and B, respectively; *p* < 0.0001, Table 3), and displayed, on average, more severe pulmonary involvement (4 [3–5] vs. 2 [1.5–4]; *p* = 0.01, Fig. 3). They were also less frequently hypertensive (*p* < 0.05), developed less frequent renal failure (*p* < 0.001) and had a lower mortality rate (7.1% and 5.2% vs. 41.2%, *p* < 0.001; Table 4 and Fig. 4). The sex ratio, lymphopaenia and D-dimer concentration were not different among the subgroups, even in younger patients.

**Table 4:** Mortality in the COVID group, univariate analysis

	Survivors (*n* = 41)	Non-survivors (*n* = 9)	*p*
Clinical data			
Age (years)	60.1 ± 2.3	78.9 ± 5.1	0.002
Height (cm)	174 ± 2	169 ± 6	0.18
Weight (kg)	88.1 ± 3.5	77.3 ± 7.8	0.20
BMI (kg/m^2^)	28.9 ± 1.0	27.3 ± 2.2	0.49
Sex ratio (M/F)	29/12	7/2	0.73
Hypertension (%)	42	89	0.01
Diabetes (%)	20	33	0.42
Smoker (%)			0.76
Never	89	79	
Former	11	19	
Current	-	2	
COPD (%)	7	44	0.003
Renal failure (%)	14	56	0.006
Cancer (%)	11	13	0.82
CT scan findings			
Thymus CT scan core 0 (%)	24	78	0.009
Lung CT scan score	3 [2–4]	4 [2–5]	0.38
Data at admission			
PaO_2_/FIO_2_	290 ± 31	264 ± 51	0.65
Haemoglobin (g/L)	12.0 ± 0.3	10.6 ± 0.7	0.06
Leukocytes (× 10^9^/L)	7.6 ± 0.6	7.9 ± 1.3	0.79
Lymphocytes (× 10^9^/L)	1.3 ± 0.2	0.4 ± 0.4	0.05
Lymphocytes (%)	18 ± 2	8 ± 3	0.009
Monocytes (× 10^9^/L)	0.63 ± 0.05	0.49 ± 0.11	0.27
Monocytes (%)	11 ± 1	7 ± 3	0.37
Fibrinogen (g/L)*	6.34 ± 0.4	7.10 ± 1.0	0.46
D-dimer (ng/mL)*	4579 ± 1158	4510 ± 2577	0.48
CRP (mg/L)	111 ± 15	206 ± 32	0.01

BMI, body mass index; COPD chronic obstructive pulmonary disease; CRP, C-reactive protein. *34 missing values. Due to multiple comparisons, significance was reached when *p* < 0.003

### Enhanced thymic function in COVID patients

**Fig. 5:**
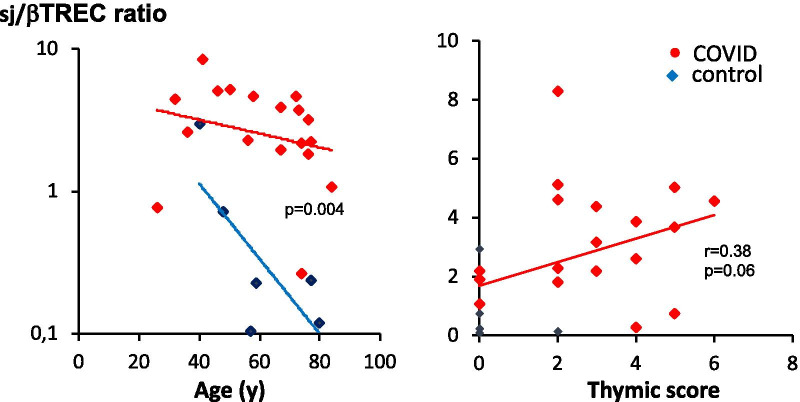
Enhanced thymic function in COVID patients. The sj/βTREC ratio, which reflects the extent of thymic output, is represented as a function of age (left) or as a function of CT scan thymic score (right). Statistical differences between groups (ANCOVA using age as a concomitant variable), nonparametric correlation Pearson’s r and associated p value (one tailed) are indicated

As expected, sjTREC values decreased as a function of age in both COVID patients and controls. sjTREC values were slightly higher in COVID individuals and correlated with the CT scan thymic score (*p* = 0.02, *n* = 24). In COVID patients, the sj/βTREC ratio was higher than in controls, particularly in older individuals, consistent with enhanced thymic production of new lymphocytes (Fig. 5). The sj/βTREC ratio was also correlated with the CT scan thymic score.

### Increased IL-7 plasma concentrations in COVID patients

**Fig. 6:**
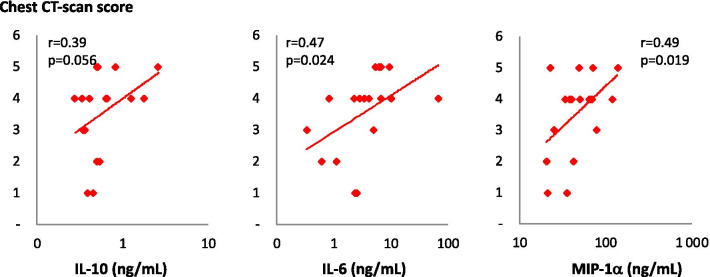
Plasma concentrations of three cytokines as a function of chest CT scan score. Plasma concentrations of IL-10, IL-6 and MIP-1α were measured in patients hospitalised for COVID (*n* = 18) and are presented as a function of chest CT scan scores. Nonparametric correlation Pearson’s r and associated p value (one tail) are indicated

**Fig. 7:**
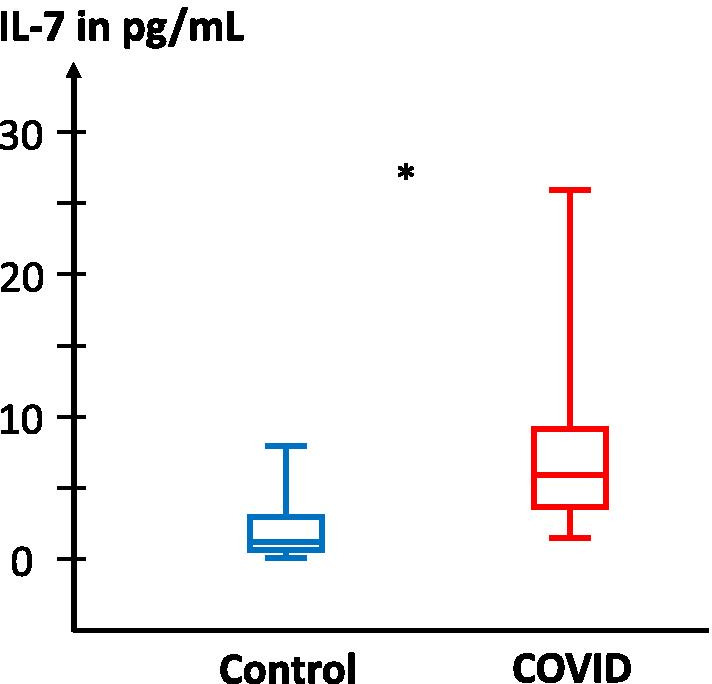
IL-7 concentrations in the plasma of control and COVID patients. IL-7 plasma concentration was measured in patients hospitalised for COVID (*n* = 18, red symbols) and controls (*n* = 6, blue symbols). Statistical significance of observed differences between groups are shown on top (Mann–Whitney *U* test)

**Fig. 8:**
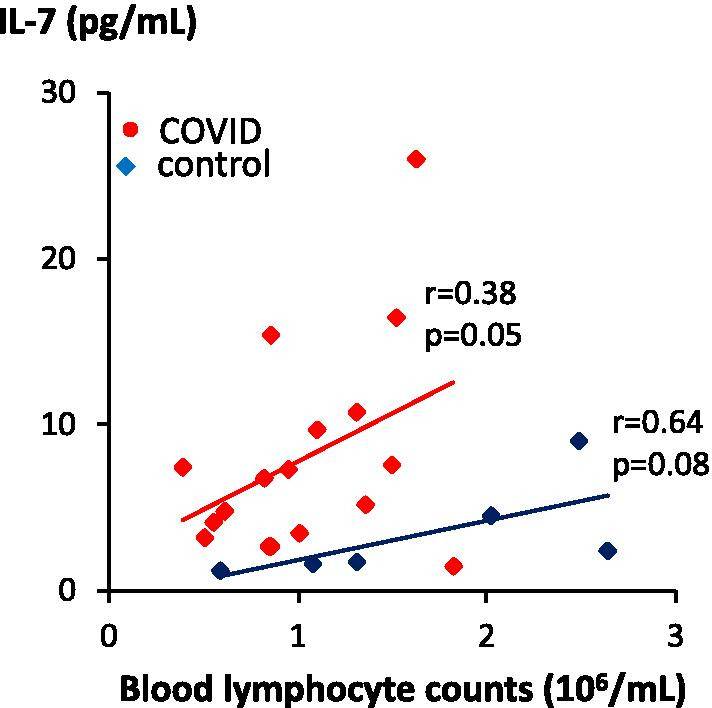
IL-7 concentrations correlated with blood lymphocyte counts. IL-7 plasma concentration was measured in patients hospitalised for COVID (*n* = 18, red symbols) and controls (*n* = 6, blue symbols) and presented as a function of blood lymphocyte counts. Nonparametric correlation Pearson’s r and associated p value (one tail) are indicated

Among the cytokines implicated in the immune response, IL-6, IL-10 and MIP-1α concentrations were correlated with COVID intensity as measured by chest CT scan (*r* = 0.47, *r* = 0.39 and *r* = 0.49; *p* = 0.024, *p* = 0.056 and 0.019, respectively; Fig. 6). Interestingly, IL-6 and IL-10 were not significantly elevated in COVID patients than in controls. In contrast, we found significantly higher concentrations of IL-7 in the plasma of COVID patients compared to uninfected controls (Fig. 7). Surprisingly, contrary to previous observations in lymphopaenic patients, IL-7 plasma levels were directly proportional to blood lymphocyte counts in both groups (Fig. 8).

## Discussion

We found that 66% of COVID patients hospitalised in our ICU displayed a surprisingly large thymus. This thymus enlargement was associated with more severe pulmonary involvement but less mortality (8.6% vs. 41.2% in the control group). These findings were not mentioned in previous reports on features associated with COVID. The emergency context in which scans of patients hospitalised in intensive care for severe forms of COVID pneumonia were performed probably contributed to underestimating the changes occurring in the thymic area. Indeed, analysis of the CT scan images of the pulmonary parenchyma requires specific settings (for example, adjustment of grey levels), which are not suitable for mediastinum analysis, causing a lack of contrast between the various mediastinal tissues.

In adults, hyperplasia of the thymus is unusual and can be observed in a limited number of pathological conditions: auto-immune diseases such as myasthenia gravis [19] and Graves-Basedow disease [20], after high-dose chemotherapy associated or not with autologous stem cell transplantation [21, 22], in lymphopaenic patients infected by human immunodeficiency virus (HIV) with maintained naïve T cell counts [23] or after antiretroviral therapy [24]. In the setting of severe T cell depletion, caused by HIV infection or cytoreductive transplant or chemotherapy regimens, thymus hyperplasia is critical for the restoration of peripheral T cell populations [22].

In patients with severe forms of COVID, severe lymphopaenia is commonly observed [15]. In our study, it was associated with overall reduced survival and inversely correlated with the intra-thymic proliferation of T cell precursors. Together, these data indicate that the increase in thymic mass observed in COVID patients is a beneficial adaptation to virus-induced lymphopaenia. Associated with increased thymic production and at least partly triggered by enhanced IL-7 levels, this adaptation appears to decline in patients older than 80 years of age, possibly contributing to the higher mortality observed in more senior patients. Severe COVID has been shown to be associated with massive infiltration of macrophages in the lungs, while recovery is dependent on T cell responses to the virus [25]. A similar progressive reduction in T cell precursor proliferation with age was previously reported in healthy HIV individuals [16] and in a systematic study of 1,000 healthy individuals showing that age and sex strongly affect thymic function [26]. Moreover, while potential genetic factors are currently under investigation in COVID patients who develop a severe form of the disease despite the absence of risk factors, it should be noted that thymopoiesis is genetically determined in both mice and healthy human adults [26, 27].

We also confirmed that the concentrations of IL-6, IL10 and MIP-1α, which are considered to be key factors of pulmonary involvement, were effectively correlated with the degree of lung involvement in COVID patients. Surprisingly, despite the already demonstrated relationship with disease intensity, IL-6 was not significantly enhanced in the COVID patient group as compared to control individuals. However, it should be noted that most of our COVID patients were not in a very severe condition, and the only patient with a very high IL-6 plasma concentration died. Moreover, our control patients cannot be considered as ‘healthy’ since they were all hospitalised in the ICU; the condition of these patients may have caused some lung inflammation, independently of the original pathological condition [28]. In contrast, we observed a significant increase in the concentration of IL-7 in the plasma of COVID patients compared to uninfected controls. A similar observation has already been reported in patients with severe COVID [29]. Unlike previous observations showing higher plasma IL-7 levels in lymphopaenic patients, plasma IL-7 levels were directly proportional to the number of blood lymphocytes in COVID patients. This observation indicates that, during acute SARS-CoV-2 infection, high plasma levels of IL-7 were not due to its low consumption by T cells, as previously suggested [30], but most likely originated from active overproduction, as previously evidenced in acute SIV infection in rhesus macaques [31]. Interestingly, IL-7 is a critical cytokine for the survival, proliferation and differentiation of immature thymocytes [32] and is a cofactor for rearrangement of the T cell receptor beta chain during early T cell development [33]. It is thus possible that the enhanced production of IL-7 in COVID patients participates in the observed thymus enlargement and increased thymic function. Unlike thymic production, the actual size of the thymus in the CT scans of patients belonging to subgroups A and B of the thymus classification was not inversely correlated with lymphopaenia. Nodular and/or minimal areas of hyperplastic thymus might be sufficient to restore an adequate number of peripheral lymphocytes.

The observation that the pulmonary involvement of COVID patients quantified by the CT scan score was significantly higher in patients with an enlarged thymus appears counter-intuitive based on the above data and hypotheses. A plausible explanation of this apparent paradox is that, in patients with an ‘activated productive’ thymus, the influx of immune cells into the infected lungs is different from that which occurs in patients with an ‘unreactive’ thymus. This hypothesis is supported by a recent study in which bronchoalveolar lavage fluid cells from COVID patients were characterised using single-cell RNA sequencing. Monocyte-derived inflammatory macrophages were found in severe COVID pneumonia, contrasting with clonal expansion of CD8 + effector T cells in mild cases, indicating a role for CD8 + T cells and the adaptive immune response in the clearance of SARS-CoV-2 [34].

This observational study, conducted in an emergency context, has some limitations. TRECs could only be evaluated in patients who were still hospitalised; some immunological investigations in the blood or bronchoalveolar lavage fluid had not been anticipated, and patient follow-up was limited in time. Prospective studies will be necessary to further characterise the role of thymic function in the control of SARS-CoV-2 infection and the involved molecular and cellular mechanisms.

## Conclusion

In response to SARS-CoV-2 infection, thymic reactivation is frequent and seems to be a good prognostic factor because it indicates the activation of compensatory mechanisms in the context of lymphopaenia, contributing to an efficient adaptive immune response in the lungs. A CT scan examination of the thymic area with appropriate settings is recommended in all COVID patients with pulmonary involvement.

## Data Availability

Should the paper be accepted, all data and materials will be available from the corresponding author (PS) and from a repository.
